# Test and Evaluation of Advertising Effect Based on EEG and Eye Tracker

**DOI:** 10.1515/tnsci-2019-0003

**Published:** 2019-04-19

**Authors:** Lingfeng Wang

**Affiliations:** 1 School of Literature and Journalism, Chongqing Technology and Business University, Chongqing, 400067, China

**Keywords:** EEG, Eye movement technology, Advertising effectiveness, Test and evaluation

## Abstract

As a means of marketing communication, advertisements have been applied in the course of enterprise operation. However, in practice, there are many problems with the implementation effect of specific advertisements, so the test and evaluation of the effectiveness of advertising have important practical and theoretical significance. Therefore, this paper uses the EEG and eye movement technology to study the EEG change and eye movement of subjects when viewing advertisements and to conduct processing and analysis of the collected EEG and eye movement indexes. It is expected to provide advertisers with valuable advertising strategies based on the analysis results of EEG change and eye movement experiment.

## 1 Introduction

In recent years, the principles and research methods of psychology have been widely applied to the study of advertising effects. Methods for measuring the psychological effects of advertising include cognitive measurement, memory measurement, visual psychometric measurement and opinion measurement. Among them, visual psychometric measurement is a kind of visual response measurement, which can be used to examine which parts of the advertisement are first, viewed when people watch advertisements, and which parts of the line are transferred to. By using the eye tracker, it is possible to record the gaze time, gaze position, and the like of the subject’s viewing of the interest area, and provide quantifiable comparative data for the advertisement effect study.

The scientific research on EEG and eye movement technology can be traced back to more than 80 years ago and its application in the medical field has been very popular. Public places are filled with various advertisements such as image advertisements, video advertisements and online advertisements. However, there is still no effective method to test and evaluate the effectiveness of this type of advertising 1.

Quantitative evaluation plays an important role in the promotion and pricing of advertising booths and has very important practical significance. EEG and eye tracking technology have been widely used in advertising researches, reading researches and space cognition researches. The existing researches not only explore the consumer’s cognitive effect on the change of physical properties of different advertisements in terms of its characteristics such as colour, location, form and celebrity endorsement effect, but also include the characteristics and appeals of audience into the scope of research, playing the application advantages of EGG and eye movement technology in accuracy and objectivity 2. Therefore, the test and evaluation of the effectiveness of advertising is of great practical and theoretical significance, which has become an important task in advertising management.

Therefore, in this study, eye movement and EEG data of 100 subjects during the experience evaluation of different pictures are collected using the Dikablis glasses eye tracker and Brain Amp64 EEG recorder. The Brain Vision Analyser software is used to process the raw data of eye movement and EEG and the correlation between the processed eye movement data, EEG data and subjective evaluation value is analysed.

## User advertising effect experience process based on EEG and eye movement technology

The factors affecting advertising effectiveness can be divided into exogenous and endogenous. Among them, the exogenous influence of advertising includes physical attributes such as size, shape, colour and location; endogenous influence refers to artificial attributes such as product involvement, motivation, existing knowledge and brand familiarity. Previous studies have found that the impact of location variables on the attention of advertising audiences cannot be ignored. Lohse used the eye movement recording method to study the eye movement characteristics of Chinese yellow pages advertisements, and found that:(1) the size effect of advertisements is very significant, people pay more attention to large advertisements and have longer fixation time; (2) size of advertisements Affect people’s ordering of advertising content, large advertisements are noticed before small advertisements; (3) the colour of advertisements also affects eye movements, and people browse time for colour advertisements longer than black and white advertisements of the same size. There are also many gaze times.

According to psychological studies, all human cognitions and perceptions are evaluative. When looking at a certain object, it judges it on the basis of good and bad, positive and negative dimensions and such evaluation and judgment information can be expressed through the physiological and psychological signals of people. Therefore, the users’ experience of advertising effectiveness includes three processes:

The first process: Eye-perception cognitive process when perceiving the evaluation object;

The second process: EEG reaction process of brain cognitive processes the evaluation subject.

The third process: The subjective evaluation process of psychological cognition processing the evaluation subject 3.

## Eye Movements Cognitive Process When Users Perceive Evaluation Advertisements

**Figure 1:**
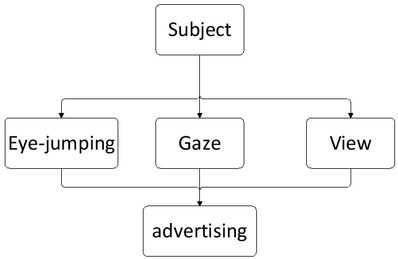
User perception eye movement cognitive process

When experiencing the advertising effect, users first search and browse the advertisement quickly through eye-jumping, then obtains the clear imaging of the advertisement through gazing and finally obtains the information expressed by the advertisement through viewing. At the same time, they also experience and evaluate the advertising effect 4. The eye movement process when experiencing the advertising effect is shown in Figure 1.

## The user’s brain recognizes the EEG response process in advertising

**Figure 2:**
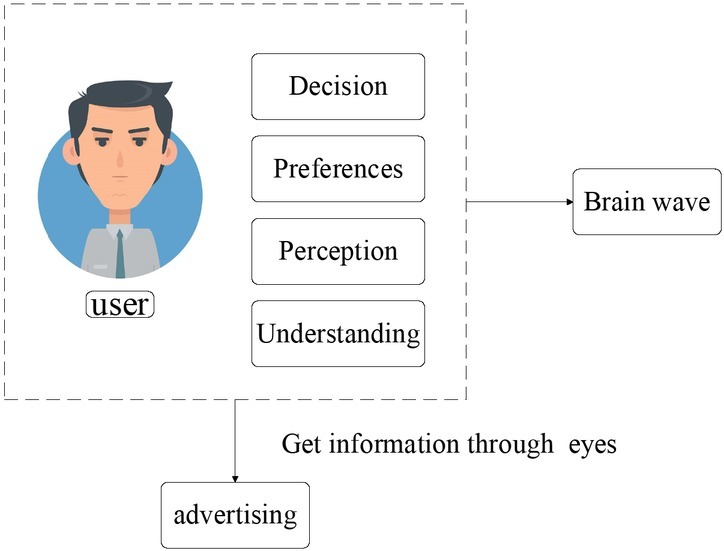
The user’s brain recognizes and handles EEG reactions during evaluation of advertisements

The users’ aesthetic cognitive experience is a process of receiving information and processing information, which can be divided into four stages: perception, understanding, preference and decision-making. The neural activities of the entire cerebral cortex, including thought, perception, cognition, decision making and mental activities, can be expressed through brain waves. The EEG response process when the users’ brain cognition processes the evaluation advertisement is shown in Figure 2.

## User appreciative cognitive process of advertising design based on eye movement technology and EEG

**Figure 3:**
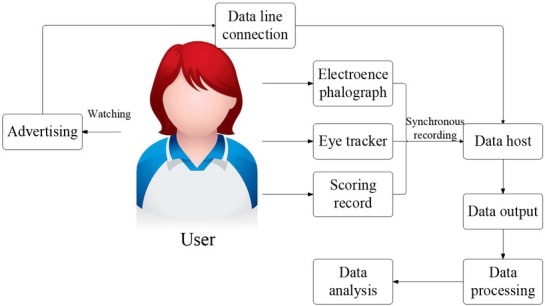
User aesthetic cognitive process of advertising design based on eye movement technology and EEG

As advertising design shifts from product-cantered to user-cantered, designers have begun to value the feeling and evaluation of users for the advertising design 5. The cognitive process of users’ aesthetics for advertising design based on eye movement technology and EEG technology is shown in Figure 3.

## Electroencephalography and eye movement experiment process Test objects

100 undergraduates, with an average age of 22 years are selected. Subjects are required to have normal uncorrected visual acuity or corrected visual acuity and are not allowed to wear coloured contact lenses during the experiment.

## Experimental advertising data and data sources

The effect picture of a tour and travel advertisement is made into the test material for evaluation and presented to the subjects. The evaluation of the test advertisement is recorded by the scoring record software; the portable eye tracker is used to synchronously record the scanning and gazing of the subjects; the electroencephalograph is used to record the brain waves of the subjects when experiencing the test evaluation; then, through the correlation analysis of the eye movement behaviour, brain waves and subjective scores with the test subjects, the psychological response and preference of subjects for the test scheme can be judged and the evaluation of the effect picture of the online tour and travel advertisement can be reflected ^[6]^.

## Experimental Schemes

The effect pictures of online tour and travel advertisements can be divided into two groups according to the experimental objective. The first group explores the effect of advertising background on the advertising effect: four pictures of three categories are selected to compare the effectiveness of advertisements with reading purposes. The second group explores the effect of advertising position on the advertising effect: three images of three categories are selected and the advertisement is placed on top or in the middle using the mapping software.

## Experimental process

The subjects enter the laboratory alone. After sitting in the seat, the EEG recorder is installed and the eye tracker is adjusted to the pre-use state. The EEG recorder and eye tracker is turned on to perform the calibration experiment and then the experiment is started. Each picture in each group is displayed for 10 seconds and subjects browse the pictures according to their daily browsing experience. When the time is up, the system will automatically switch to the next one. After the completion of the EEG experiment and eye movement experiment, the subjects are required to fill out questionnaires to further understand other information.

## Establishment of User Evaluation Model Based on EEG and Eye Movement Techniques

Let the test scheme set as *C* = {*c*1,*c*2,*c*3,...*cm*} and the subject set is *B* = {*b*1,*b*2,*b*3,...*cn*} . In the formula: m represents the number of test schemes; n represents the number of subjects.

## Eye movement test evaluation model of user advertising effect

The eye tracker is used to synchronously record the eye movement data of the subjects after viewing different advertisement pictures and the corresponding eye movement test index data are extracted, as is shown in Formula 1:

(1) $$Y\left(bi,ci,F\right)=\left\{\begin{array}{l} y\left(b1,\;c1,\;F\right),y\left(b1,\;c2,\;F\right),\ldots ,\;y\left(b1,\;cm\;F\right)\\ y\left(b2,c1,F\right),y\left(b2,c2,F\right),\ldots ,y\left(b2,cm,F\right)\\ \;\;\;\;\;\;\;\;\;\;\;\;\;\;\;\;\;\;\;\;\;\;\;\;\;\;\;\;\;\;\;\;\;\;\;\;\;\;\;\vdots \\ y\left(bn,c1,F\right),y\left(bn,c2,F\right),\ldots ,y\left(bn,cm,F\right) \end{array}\right\}$$

In the formula: *Y*(*bi, cj, F*)is the eye movement test index data of the subject *bi* for the test scheme *cj*. *F =* {*f1, f2,...,fg*} is the eye movement test index, including the number of gazing in the area of interest and gazing time 7. The comprehensive value of the eye movement data of all the test schemes j is:

(2) y(b1−n,cj,F)=y(1,cj,F)+y(2,cj,F)+y(n,cj,F)n

The comprehensive value of the eye movement data for each scheme is:

(3) $$Y^{T}=\left[y_{c1},y_{c2},\ldots ,y_{cm}\right]$$

## EEG test evaluation model for user advertising effectiveness

The electroencephalogram is used to synchronously record the EEG data of 64 electrodes in the cerebral cortex in sequence when the subjects are viewing different advertisement pictures, as is shown in formula 4.

(4) $$D\left(bi,cj,G\right)=\left\{\begin{array}{c} d\left(b1,c1,G\right),\;d\left(b1,c2,G\right),\ldots ,d\left(b1,cm,G\right)\\ d\left(b2,c1,G\right),\;d\left(b2,c2,G\right),\ldots ,d\left(b2,cm,G\right)\\ \vdots \\ d\left(bn,c1,G\right),\;d\left(bn,c2,G\right),\ldots ,d\left(bn,cm,G\right) \end{array}\right\}$$

In the formula: *D*(*bi, cj, G*) is the EEG data of the subject *bi* for the test scheme *cj*; *G =* (*g*1*, g*2*,...,gi,....,g*64) represents the EEG data of *i*-th electrode. The comprehensive value of EEG data of all test schemes j is:

(5) d(b1−n,cj,G)=d(1,cj,G)+y(2,cj,G)+y(n,cj,G)n

The comprehensive value of the eye movement data for each scheme is:

(6) $$D^{T}=\left[d_{c1},d_{c2},\ldots ,d_{cm}\right]$$

## Eye movement technology and EEG comprehensive evaluation model for advertising effectiveness

The eye tracking data and brain waves of the subjects can be recorded as

(7) $$Z=\left(Y,F\right)=\left[\begin{array}{c} y_{c1},d_{c1}\\ y_{c2},d_{c2}\\ \vdots \\ y_{cm},d_{cm} \end{array}\right]$$

## User Experience Test Results and Evaluation of Online Travel Advertising

## User’s eye movement data processing result and evaluation of advertising effectiveness

The gazing time refers to the time from the display of the online travel advertisement to the judgment of the subject. Substituting the experimental data into formula (1), formula (2) and formula (3), the average gazing time of all subjects experiencing the effect picture of the advertisement can be obtained. The number of gazing refers to the number of gazing from the display of the effect picture of advertisement to the judgment of the subject. Substituting the experimental data into formula (1), formula (2) and formula (3), the average number of gazing of all subjects experiencing the effect picture of the advertisement can be obtained 8.

## Impact and Evaluation Analysis of Advertising Background Type on Advertising Effectiveness

There are only average value of the sum of the duration of gazing points and number of visits of online travel advertisements and of the subjects in the page of pictures only, text only and combination of pictures and texts (see Table 1).

**Table 1:** Average value of mean time (ms) and number of visits for each interest area under different background types

Background type	Images	Texts	Images + Tests	Fiction
Watching time (average)	874	532	573	735
Number of visits	21	8	15	4

The average value gazing and the times are high when the background of advertisements is picture only; in the novel page, although the number of visits is small, the gazing time is long; in the text-only page, the visit time is short. The colour contrast of the background and the advertisement can easily give rise to different attention to the advertisement of the subjects. This is particularly obvious in the novel page. Although the control method is used in this experiment, requiring participants to carefully read the content of the novel, the viewing time of the advertisement area on the page ranks the second. At the same time, the attention brought by the contrasting difference is reflected in the background of picture only. Compared with the novel page, the advertisement in the background of pictures receives higher degree of attention. Since pictures contain less information compared with other texts, the subjects have more time to pay attention to the advertisement. In terms of the number of visits, pages with both texts and pictures receive more visits than novel pages. In the novel page, it is easy to distinguish between the advertising area and the text area. Those who are not interested in advertisements will automatically choose to read in other areas and those who are interested in advertisements will read these advertisements carefully [9, 10, 11].

## Impact of Advertisement Position on Advertising Effectiveness

The impact of ad position on the advertising effectiveness is not significant in the picture background, but from the perspective of the gazing time and number of gazing, when the ad is at the top of the page, the viewing time is long and the average time is large, which may be caused by Chinese people’s reading habit from top to the bottom (as is shown in Table 2).

**Table 2:** Average of the time of watching and the number of visits in each area of interest in different locations

Background type	Interest area location			
	Upper apex		Centre	
	Watching time	Number of visits	Watching time	Number of visits
Images	1012	15	892	19
Texts	472	6	371	6
Images + Tests	643	12	353	5

## EEG data processing results and evaluation of user advertising effectiveness

The original signals collected in the experiment contain spontaneous EEG signals, evoked EEG signals and other physiological interference signals. The evoked EEG signals contain the evaluation information of users for the advertising effectiveness. Therefore, it is necessary to extract and analysed the EEG signals. The Brain Vision analyser software is used to extract the evoked EEG signals of 64 electrodes. After extracting and analysing the evoked EEG signals of 64 electrodes, the energy distribution of the EEG evoked by each effect picture of advertisement can be observed more intuitively 12. The electrodes of the cognitive evaluation of aesthetic experience in event-related potentials (ERP) include: FZ, FC1, F1, F2, F3, F4, F5, F6, F7 and F8. When users are watching two groups of different experimental schemes, the ERP data of the above ten electrodes will be different. Therefore, it is necessary to compare and analyse the evoked EEG data of these ten electrodes. It can be found through examining the EEG comparative analysis diagram that the peak value of the evoked brain wave is different when users are viewing two groups of different schemes, as is shown in Table 3.

**Table 3:** The peaks of brain waves induced in different electrodes in two groups of protocols

Induced electrode	FZ	FC1	F1	F2	F3	F4	F5	F6	F7	F8
Scheme 1	2.5	4.9	3.5	3.3	1.0	2.2	3.9	3.0	1.9	1.2
Scheme 2	7.3	4.9	3.7	7.9	3.2	4.2	4.9	5.1	1.0	1.9

**Correlativity analysis of experimental results of advertising users’ evaluation under electroencephalography and eye movement technology**

The correlation analysis is conducted on the eye movement gazing time (Y1), eye movement gazing times (Y2) and the peak value of the evoked EEG FZ, FC1, F1, F2, F3, F4, F5, F6, F7 and F8 is performed, obtaining the analysis results of the correlation coefficient, as is shown in Table 4.

**Table 4:** Relationship between EEG and eye movement

	Y1	Y2	FZ	FC1	F1	F2	F3	F4	F5	F6	F7	F8
Y1	1											
Y2	0.84	1										
FZ	0.98	0.79	1									
FC1	0.95	0.71	0.69	1								
F1	0.89	0.97	0.83	0.97	1							
F2	0.72	0.84	0.75	0.69	0.77	1						
F3	0.93	0.93	0.83	0.93	0.46	0.93	1					
F4	0.95	0.91	0.86	0.67	0.74	0.69	0.91	1				
F5	10.89	0.95	0.97	0.88	0.98	0.63	0.71	0.93	1			
F6	0.82	0.89	0.92	0.93	0.79	0.69	0.90	0.99	0.96	1		
F7	0.79	0.83	0.85	0.96	0.78	0.89	0.80	0.93	0.89	0.93	1	
F8	0.69	0.73	0.99	0.85	0.83	1	0.79	0.98	0.99	0.90	0.90	1

It can be seen through the correlation analysis that the correlation coefficient of physiological and psychological indexes for the test and evaluation of the design scheme of online travel advertisements is positive. Except that the correlation coefficient of electrodes F1 and F3are 0.46 respectively, all other correlation coefficients are greater than 0.6, showing a significant positive correlation. It can be seen that the evaluation results for the design scheme of all evaluation indexes are all relatively good.

## Conclusions

At present, the research on advertising effects mainly focuses on traditional paper media and web pages. However, with the advent of the Web 3.0 era, mobile phones have become the medium of media that cannot be ignored. According to CNNIC data, as of June 30, 2013, the number of mobile Internet users in China has reached 464 million. As a carrier of information dissemination between merchants and consumers, mobile APP advertising has become an important means for businesses to expand their influence. The advertising forms mainly include banner advertisements, full-page advertisements, barrage advertisements, etc., which have the characteristics of wide spread and various forms of expression. Banner advertisements have been widely used in mobile APP due to their small size and long duration.

This paper designs two groups of experimental schemes for advertisements and conducts the user test and evaluation study of the advertising effectiveness. This paper considers the psychological and physiological feelings of users in the advertisement experience process and proposes the user experience test and evaluation method of the advertisement effectiveness based on EEG technology and eye movement technology. The experimental research results show that there is a significant positive correlation between evaluation indexes such as the EEG and eye movement in the user experience test and evaluation of advertisements and the evaluation results in each dimension can be mutually verified, which provides richer evaluation indexes for the user experience test and evaluation of advertising design and effectively reduces the capital investment risk in the late stage caused by errors in the scheme selection in the previous advertising design stage.
